# Person-Zentrierung in der stationären Altenhilfe: Eine qualitative Dokumentenanalyse von Leitbildern

**DOI:** 10.1007/s00391-025-02443-3

**Published:** 2025-05-30

**Authors:** Anna Louisa Hoffmann-Hoffrichter, Mike Rommerskirch-Manietta, Bernhard Holle, Rebecca Palm, Martina Roes

**Affiliations:** 1 Deutsches Zentrum für Neurodegenerative Erkrankungen e. V. (DZNE), Stockumer Str. 12, 58453 Witten, Deutschland; 2 Fakultät für Gesundheit, Universität Witten/Herdecke (UW/H), Witten, Deutschland; 3 Fakultät VI Medizin und Gesundheitswissenschaften, Carl von Ossietzky Universität Oldenburg, Oldenburg, Deutschland

**Keywords:** Person-zentrierte Versorgung, Leitbilder, Dokumentenanalyse, Demenz, Wohnbereiche, Person-centred care, Mission statements, Document analysis, Dementia, Care units

## Abstract

**Hintergrund:**

Die Umsetzung von Person-Zentrierung in die Versorgungspraxis von Pflegeeinrichtungen kann durch Leitbilder, die u. a. eine klar kommunizierte Vision adressieren, positiv beeinflusst werden. Es ist bekannt, dass Pflegeeinrichtungen mit Demenzwohnbereich im Vergleich zu solchen mit traditionellem Wohnbereich Fortbildungen oder eine Expert:in zu Person-Zentrierung vorhalten können. Unklar ist, wie sich Leitbilder dieser Pflegeeinrichtungen hinsichtlich ihrer Formulierung von Person-Zentrierung unterscheiden. Das Ziel ist es zu untersuchen, wie person-zentrierte Versorgung in Leitbildern von Pflegeeinrichtungen, die verschiedene Wohnbereiche vorhalten, beschrieben wird.

**Methoden:**

Es wurde eine Dokumentenanalyse von Leitbildern deutscher Pflegeeinrichtungen eines Datensatzes der Survey-Studie zur besonderen stationären Versorgung von Menschen mit Demenz in Pflegeeinrichtungen (BeStaDem-Survey-Studie) durchgeführt. Die Pflegeeinrichtungen, die die Leitbilder zur Verfügung stellten, hielten verschiedene Wohnbereiche vor, darunter traditionelle Wohnbereiche sowie Demenzwohnbereiche. Die Leitbilder wurden mittels inhaltlich-strukturierender Inhaltsanalyse deduktiv-induktiv analysiert.

**Ergebnisse und Diskussion:**

Es wurden 60 Leitbilder verschiedener Pflegeeinrichtungen analysiert. Dabei wurden Hauptkategorien, die Aspekte der person-zentrierten Versorgung beinhalten, identifiziert. Insbesondere Leitbilder von Pflegeeinrichtungen mit Demenzwohnbereichen beschreiben Kombinationen zentraler Aspekte zu person-zentrierter Versorgung, die Leitbilder von Pflegeeinrichtungen mit einem traditionellen Wohnbereich ohne demenzspezifische Ausrichtung nicht beschreiben. In Leitbildern wird häufig eine Variation verschiedener Verständnisse bzw. Konzepte deutlich. Damit Pflegeeinrichtungen ihr Leitbild person-zentriert ausrichten können, bedarf es einer umfassenden Auseinandersetzung mit dem person-zentrierten Ansatz und eines Entwicklungsprozesses, der alle Mitarbeitenden einschließt.

## Abstract (en)

**Background:**

Mission statements that address a clearly communicated vision can positively influence the implementation of person-centred care in nursing homes. It is known that nursing homes with a dementia care unit can provide further training or an expert on person-centredness compared to those with a traditional care unit. It is unclear how the mission statements of these nursing homes differ in terms of their wording of person-centredness. The aim is to investigate how mission statements of nursing homes that provide different types of care units describe person-centred care.

**Methods:**

We conducted a document analysis of mission statements from German nursing homes from a dataset of the survey study on specialized care for people living with dementia in nursing homes (BeStaDem Survey Study). The nursing homes that provided the mission statements had different care units, including traditional care units and dementia care units. The mission statements were deductively-inductively analysed using content analysis.

**Results and conclusions:**

We analysed 60 mission statements of different nursing homes and identified main categories that include aspects of person-centred care. In particular, mission statements of nursing homes with dementia care units describe combinations of central aspects of person-centred care that mission statements of nursing homes with a traditional care unit without dementia-specific focus do not describe. The variation of different understandings or concepts often becomes evident in mission statements. In order for nursing homes to align their mission statement with a person-centred approach, a comprehensive understanding of person-centred care and a development process that includes all employees, is required.

## Hintergrund und Fragestellung

In Deutschland lebten Ende 2021 circa 1,8 Millionen Menschen mit einer demenziellen Erkrankung [6]. In langzeitstationären Pflegeeinrichtungen zählt diese Personengruppe mit 51,8 % zu der größten Gruppe dort lebender Menschen [14, 26]. Das macht eine Versorgung notwendig, die die Lebensqualität und das Wohlbefinden gleichwohl der Personen mit Demenz als auch der Personen, die diese Versorgung erbringen, aufrechterhält. Ergebnisse unterschiedlicher Studien zeigen, dass person-zentrierte Versorgung die Lebensqualität und das Wohlbefinden begünstigt [4]. Darunter wird nach der Konzeption Kitwoods [18] ein Ansatz verstanden, der die Person, ihre Identität, Autonomie und Präferenzen in den Mittelpunkt der Versorgung stellt [18]. Die Umsetzung dieses Ansatzes wird im nationalen Expertenstandard zur Beziehungsgestaltung von Menschen mit Demenz [10] gefordert.

Backman et al. [3] identifizierten in ihrer Studie verschiedene Barrieren zur Umsetzung von person-zentrierter Versorgung, darunter das Fehlen einheitlicher Werte, Prioritäten und Prozesse innerhalb des Teams. Damit der person-zentrierte Ansatz in einer Organisation der stationären Altenhilfe nachhaltig und wertebasiert umgesetzt werden kann, ist zunächst eine Vision vonnöten, die in der jeweiligen Organisation gelebt wird [22, 25]. Eine Vision fasst die strategischen Ziele einer Organisation zusammen und ist neben der Mission und den Werten ein Bestandteil des organisationalen Leitbildes [19]. Als das am häufigsten verwendete Managementinstrument [1] werden Leitbilder nach außen hin als Visitenkarte der Pflegeeinrichtungen verstanden [17]. Theoretisch leitet sich aus dem Leitbild das Handeln der Mitarbeitenden ab, um die formulierten Ziele zu erreichen [22]. Organisationen können in einem Leitbild das Einrichtungs- und Pflegeleitbild vereinen, aber auch zwischen Einrichtungs- und Pflegeleitbild unterscheiden [17]. Auch wenn Leitbilder als ein Teil der Strukturqualität in den rechtlich bindenden Maßstäben und Grundsätzen zur Qualität in der vollstationären Pflege nach § 113 des Elften Sozialgesetzbuches (SGB XI) aufgeführt werden, korrespondieren Inhalte, die im Leitbild beschrieben werden, nicht zwangsläufig mit der alltäglichen Pflegepraxis der jeweiligen Organisation [16].

Bis dato wurde für Deutschland untersucht, inwiefern Regularien zu person-zentrierter Versorgung in der stationären Altenhilfe Ziele und Werte der Leitbilder von Pflegeeinrichtungen widerspiegeln [15]. Es wurde nicht untersucht, wie person-zentrierte Versorgung darüber hinaus in Leitbildern beschrieben wird. Darüber hinaus fanden Bergmann et al. [5] heraus, dass sich Demenzwohnbereiche im Vergleich zu traditionellen Wohnbereichen darin kennzeichnen können, dass sie u. a. eine Pflichtfortbildung zu Person-Zentrierung für alle Mitarbeitenden vorhalten oder eine/einer der Mitarbeitenden Expert:in für person-zentrierte Pflege ist. Unklar blieb jedoch, inwiefern sich Pflegeeinrichtungen mit einem Demenzwohnbereich hinsichtlich der person-zentrierten Ausrichtung ihres Leitbildes von Pflegeeinrichtungen unterscheiden, die einen traditionellen Wohnbereich vorhalten.

Daraus ergeben sich die folgenden Fragestellungen:

- Wie wird person-zentrierte Versorgung in Leitbildern von Pflegeeinrichtungen beschrieben?
- Wie unterscheiden sich Leitbilder von Pflegeeinrichtungen, die einen Demenzwohnbereich vorhalten, im Vergleich zu Pflegeeinrichtungen, die einen traditionellen Wohnbereich vorhalten?

## Studiendesign und Untersuchungsmethoden

Diese Studie ist eine qualitative Dokumentenanalyse [7, 12] von Leitbildern aus Pflegeeinrichtungen in Deutschland. Bei einer Dokumentenanalyse handelt es sich um ein systematisches Verfahren mit dem Ziel, Dokumente zu prüfen und auszuwerten [7]. Dabei sind Dokumente existierendes Material der menschlichen Praxis, zu denen auch Leitbilder zählen [12]. Als Analyseeinheit wurden sowohl Pflegeleitbilder als auch Trägerleitbilder der Pflegeeinrichtungen festgelegt.

### Datenerhebung

Die Leitbilder von Pflegeeinrichtungen, die in dieser Studie genutzt werden, stammen aus einem Datensatz der Survey-Studie zur besonderen stationären Versorgung von Menschen mit Demenz in Pflegeeinrichtungen (BeStaDem-Survey-Studie), einer nationalen Querschnittstudie mit einem Sample von 134 Pflegeeinrichtungen in Deutschland [5, 13].

In die BeStaDem-Survey-Studie wurden vollstationäre Pflegeeinrichtungen mit Versorgungsvertrag nach Sozialgesetzbuch (SGB) XI eingeschlossen. Aus einer Liste [23] aller Pflegeeinrichtungen in Deutschland wurden, stratifiziert nach Bundesland und Vorhandensein eines Demenzwohnbereichs, randomisiert Pflegeeinrichtungen gezogen.

Im Rahmen standardisierter Telefoninterviews in der BeStaDem-Survey-Studie von Juni bis Dezember 2020 wurden die Teilnehmenden gebeten, im Nachgang ihr Pflegeleitbild per E‑Mail an das Studienteam zu senden [5].

Das Ziel der BeStaDem-Survey-Studie war es, eine Typologie von Wohnbereichen in Pflegeeinrichtungen zu entwickeln. Im Rahmen standardisierter Telefoninterviews wurden Daten erhoben; diese wurden mittels Faktorenanalyse gemischter Daten („factor analysis of mixed data“, FAMD) und hierarchischer Cluster-Analyse analysiert. Im Ergebnis konnte die Typologie grundsätzlich zwischen Demenzwohnbereichen und traditionellen Wohnbereichen unterscheiden [5]. In Tab. 1 sind die Unterschiede, die diese Wohnbereiche kennzeichnen können, zusammengefasst. Diese Zuordnung der Wohnbereiche wurde für die Zuordnung der Leitbilder in der qualitativen Dokumentenanalyse verwendet.

**Tab. 1:** Beschreibung der Wohnbereichstypen. (In Anlehnung an Bergmann et al. [5])

Wohnbereich	Mögliche Merkmale
Demenzwohnbereich	*Selbstbezeichnung als Demenzwohnbereich* *Speziell für Menschen mit Demenz gebaut* *Aus- und Zugangskontrollen* *Höherer Prozentsatz an Menschen mit Demenz* *Mit Kostenträgern wurden vereinbart* – Ein pflegefachlicher Schwerpunkt für Menschen mit Demenz – Im Vergleich zu anderen Wohnbereichstypen ein höherer Personalschlüssel für Pflegefachkräfte – Eine besondere Vergütungsvereinbarung – Aufnahmekriterien für Bewohner:innen
Traditioneller Wohnbereich	*Nicht demenzspezifisch* *Nicht speziell für Menschen mit Demenz gebaut* *Keine Aus- und Zugangskontrollen* *Keine Aufnahmekriterien* *Niedriger Prozentsatz an Menschen mit Demenz im Vergleich zum Demenzwohnbereich*

Die Ethikkommission der Deutschen Gesellschaft für Pflegewissenschaft erteilte im Oktober 2018 das ethische Clearing für die BeStaDem-Survey-Studie (Antrag Nr. 18-016).

### Datenaufbereitung

Von 134 haben 60 Pflegeeinrichtungen ihr Leitbild zur Verfügung gestellt. Für die vorliegende Studie wurden alle Leitbilder gemäß Bowen [7] geprüft: Die Dokumente wurden beispielsweise dahingehend geprüft, ob sie tatsächlich von der jeweiligen Pflegeeinrichtung bereitgestellt wurden. Anhand der Überschrift und der Inhalte der Dokumente wurde geprüft, ob es sich um die Leitbilder handelt und der Inhalt in sich konsistent ist. In dem Fall, dass Pflegeeinrichtungen ihr gesamtes Qualitätsmanagementhandbuch zur Verfügung gestellt haben, wurden die Dokumente für die Analyse so aufbereitet, dass lediglich das Leitbild analysiert wurde. Um Kontextinformationen zu erhalten, wurden zu jedem Leitbild zunächst folgende Informationen in einem Protokoll festgehalten:

1. Demenzwohnbereich oder traditioneller Wohnbereich, dem die Pflegeeinrichtung in den Ergebnissen der BeStaDem-Survey-Studie zugeordnet wurde,
2. Leitbildart (Pflegeleitbild, Trägerleitbild),
3. Funktion der Person, die das Leitbild erstellt hat,
4. Datum der Erstellung,
5. Zusammenfassung des Leitbilds.

### Datenanalyse

Die Leitbilder der Pflegeeinrichtungen wurden in MAXQDA 2022 (VERBI Software, Berlin, Deutschland) [27] überführt und gemäß der inhaltlich-strukturierenden Inhaltsanalyse nach Kuckartz und Rädiker [21] deduktiv-induktiv durch Autorin 1 und Autor 2 analysiert.

Zunächst wurden die Leitbilder hinsichtlich des Vorhandenseins von Regularien zu person-zentrierter Versorgung von Menschen mit Demenz analysiert. Diese Datenanalyse und Ergebnisse wurden an anderer Stelle publiziert [15]. Für diese Studie wurden anhand des person-zentrierten Ansatzes nach Kitwood [18] deduktiv Kategorien hergeleitet und für die Kodierung des Materials genutzt. Bei Bedarf wurden die deduktiven Kategorien induktiv ergänzt. Subkategorien und Kodes aus dem Datenmaterial wurden induktiv gebildet.

Um mit den Daten vertraut zu werden, haben Autorin 1 und Autor 2 alle Leitbilder gelesen und Memos erstellt. Autorin 1 erstellte ein Kategoriensystem. Dieses umfasst Hauptkategorien und deduktive Kategorien, die Aspekte des person-zentrierten Ansatzes nach Kitwood [18] enthalten (Tab. 3). Um die Anwendbarkeit des Codebaumes zu überprüfen, haben Autorin 1 und Autor 2 anhand des Kategoriensystems unabhängig voneinander 10 Leitbilder kodiert. Die Kodierungen wurden besprochen und diskutiert. Daraufhin wurden die Kategoriedefinitionen angepasst. Anschließend wurden alle Leitbilder unabhängig voneinander kodiert. In regelmäßigen Treffen wurde das Kategoriensystem sukzessive präzisiert. Abweichende Kodierungen wurden diskutiert. Kategorien ohne Konsens wurden im erweiterten Studienteam diskutiert, bis ein Konsens erreicht wurde. Anschließend wurden alle Leitbilder kodiert. Während des Analyseprozesses wurden die Leitbilder der Pflegeeinrichtungen unterschiedlicher Wohnbereichstypen verglichen und kontrastiert. Alle Kategorien wurden mit ihren Subkategorien und den Kontrastierungen der einzelnen Wohnbereichstypen in thematischen Memos festgehalten und weiterentwickelt. Zuletzt wurde eine kategorienbasierte Analyse entlang der Kategorien sowie Übersichten und Vergleiche der Wohnbereichstypen vorgenommen.

## Ergebnisse

Von den 60 Pflegeeinrichtungen, die ihr Leitbild zur Verfügung stellten, halten 33 einen Demenzwohnbereich und 27 einen traditionellen Wohnbereich vor. 43 Pflegeeinrichtungen stellten ihr Pflegeleitbild zur Verfügung, 17 Pflegeeinrichtungen das Leitbild ihres Trägers [15]. Weitere Kontextinformationen sind in Tab. 2 aufgeführt.

**Tab. 2:** Kontextinformation der Leitbilder

Kontextinformation	Häufigkeit (%), *n* = 60
*Funktion der erstellenden Person*	
Qualitätsmanagement	14 (23,33)
Qualitätsmanagement und Leitungsperson	2 (3,33)
Leitungsperson(en)	7 (11,67)
Pflegebesprechung, Arbeitskreis, Mitarbeitende	3 (5,00)
Keine Angabe	34 (56,67)
*Datum Erstellung/Prüfung/Freigabe*	
Vor 2010	5 (8,33)
Von 2010–2014	6 (10,00)
Von 2015–2019	22 (36,67)
2020 und später	6 (10,00)
Keine Angabe	21 (35,00)

Insgesamt benennen 4 Leitbilder explizit den person-zentrierten Ansatz nach Kitwood, darunter ein Leitbild einer Pflegeeinrichtung mit einem traditionellen Wohnbereich und 3 Leitbilder von Pflegeeinrichtungen mit einem Demenzwohnbereich.

Die Ergebnisse umfassen folgende Hauptkategorien: Bewohner:in als Person, person-zentriertes Handeln, person-zentrierte Outcomes sowie kulturelles Verständnis. In Tab. 3 sind die Hauptkategorien mit den Kategorien aufgeführt, die Aspekte der person-zentrierten Versorgung abbilden. Mindestens ein Aspekt person-zentrierter Versorgung konnte in allen Leitbildern identifiziert werden. In Leitbildern von Pflegeinrichtungen mit Demenzwohnbereichen wurde in der Analyse deutlich, dass sie eine Kombination von Aspekten person-zentrierter Versorgung adressieren. Vier Leitbilder von Pflegeeinrichtungen mit einem Demenzwohnbereich adressieren die/den Bewohner:in als Person mit seiner Persönlichkeit, thematisieren Ressourcen und Identität. Gleichzeitig wurde in keinem der Leitbilder von Pflegeeinrichtungen mit einem traditionellen Wohnbereich dieser Aspekt adressiert.

**Tab. 3:** Kategoriensystem mit Hauptkategorien und Kategorien

Hauptkategorie	Kategorien
Bewohner:in als Person	Ressourcen
	Persönlichkeit
	Identität
Person-zentriertes Handeln	Bewohner:in im Zentrum des Handelns
	Die/den Bewohner:in als Person anerkennen
	Individueller Umgang
	Präferenzorientierter Umgang
	Autonomie und Selbstbestimmung ermöglichen
	Inklusion
	Positive Arbeit an der Person
	Auf psychische Bedürfnisse eingehen
	Beziehungsgestaltung
Person-zentrierte Outcomes	Wohlbefinden
	Lebensqualität
	Zufriedenheit
	Geborgenheit
	Erhalt von Personsein
	Erhalt von Selbstbestimmung
Kulturelles Verständnis	Grundlagen von Haltung
	Haltung der Mitarbeitenden
	Atmosphäre

Im Folgenden werden inhaltliche Unterschiede der Leitbilder von Pflegeeinrichtungen mit den 2 Wohnbereichstypen für jede Hauptkategorie beschrieben.

### Bewohner:in als Person

Während Leitbilder von Pflegeeinrichtungen mit einem traditionellen Wohnbereich keine Attribute, die die/den Bewohner:in als Person kennzeichnen, beschreiben, wurden in 4 Leitbildern von Pflegeeinrichtungen mit Demenzwohnbereichen Attribute identifiziert. Diese Attribute umfassen individuelle Merkmale, wie Ressourcen, die Persönlichkeit und Identität, sowie den für das Personsein wichtigen Aspekt der Reziprozität.

Die Ressourcen der/des Bewohner:in werden beschrieben als Merkmal, die sie/ihn mit seinen Fähigkeiten und Möglichkeiten zu einer einmaligen Person machen. Die Persönlichkeit der/des Bewohner:in wird als Einmaligkeit und Einzigartigkeit der in der Pflegeeinrichtung lebenden Personen und ihrem Erleben beschrieben. In 3 Leitbildern von Pflegeeinrichtungen mit Demenzwohnbereich wird die Identität als von Herkunft und Lebensgeschichte geprägt beschrieben; die wird erst durch soziale Interaktion erfahren und macht die Person zu einem einmaligen Individuum:

„Erst im Miteinander erfährt der Mensch seine unverwechselbare Identität.“ (Pflegeeinrichtung 112, Demenzwohnbereich)

Aus diesen Eigenschaften wird in den Leitbildern, die die/den Bewohner:in als Person beschreiben, person-zentriertes Handeln des Pflegepersonals abgeleitet.

### Person-zentriertes Handeln

Person-zentriertes Handeln fasst alle Aspekte der Leitbilder, in denen die/der Bewohner:in in das Zentrum der Interaktion mit Pflegenden gestellt wird, zusammen. In den Leitbildern von Pflegeeinrichtungen beider Wohnbereichstypen wird beschrieben, dass person-zentriertes Handeln zum Ziel hat, das Personsein der/des Bewohner:in zu stärken und zu erhalten. Hierbei werden in Leitbildern beider Wohnbereichstypen verschiedene Aspekte beschrieben: die/den Bewohner:in als Person anerkennen, der individuelle Umgang, der präferenzorientierte Umgang, das Ermöglichen von Autonomie und Selbstbestimmung, die Inklusion, die positive Arbeit an der Person, auf psychische Bedürfnisse eingehen sowie die Beziehungsgestaltung. Im Kontext der Beziehungsgestaltung adressiert ein Leitbild einer Pflegeeinrichtung mit Demenzwohnbereich die Validation:

„Um die individuelle Lebenswelt der Menschen mit Demenz besser verstehen zu können, haben wir uns der Validation verschrieben.“ (Pflegeeinrichtung 109, Demenzwohnbereich)

In Leitbildern von Pflegeeinrichtungen beider Wohnbereichstypen wird eine Variation verschiedener Verständnisse von Handeln deutlich: Leitbilder beschreiben sowohl, dass die Pflegeeinrichtung selbstbestimmtes Handeln der/des Bewohner:in berücksichtigt und ermöglicht, als auch den Anspruch einer Versorgung verfolgt, die die Selbstständigkeit der/des Bewohner:in aufrechterhält. Am häufigsten wird diese Variation in Leitbildern von Pflegeeinrichtungen mit Demenzwohnbereich deutlich, z. B.:

„Wir berücksichtigen ihre Biografie, Religion und Gewohnheiten und bewahren ein selbst bestimmtes Leben. […]. Auf der Basis einer ganzheitlichen, aktivierenden und dokumentierten Pflege orientieren wir uns am Pflegeprozess. Gemeinsam erkennen wir dabei die vorhandenen Fähigkeiten der Bewohner und fördern den Erhalt ihrer Selbstständigkeit.“ (Pflegeeinrichtung 66, Demenzwohnbereich)

In 4 Leitbildern von Pflegeeinrichtungen mit einem Demenzwohnbereich, die zugleich die/den Bewohner:in als Person charakterisieren, wird eine konzeptionelle Variation nicht deutlich.

### Person-zentrierte Outcomes

In den Leitbildern werden Vorhaben und Ziele person-zentrierter Outcomes, wie das Wohlbefinden, die Lebensqualität sowie die Zufriedenheit und Geborgenheit der/des Bewohner:in, aber auch der Erhalt des Personseins beschrieben. Spezifisch für Leitbilder von Pflegeeinrichtungen mit Demenzwohnbereich ist, dass sie das Outcome des Erhalts von Selbstbestimmung als pflegerisches Vorhaben beschreiben, z. B.:

„Wir wollen die Selbstbestimmung, die Entscheidungsfreiheit und die Lebenszufriedenheit jedes Menschen fördern und erhalten.“ (Pflegeeinrichtung 60, Demenzwohnbereich)

### Kulturelles Verständnis

In den Leitbildern werden Aspekte des kulturellen Verständnisses von Pflege beschrieben, die Fundamente von und Aspekte der Haltung der Mitarbeitenden sowie der Atmosphäre in den Pflegeeinrichtungen beinhalten.

Zu Aspekten, die Fundamenten von Haltung oder Einstellungen zugeordnet werden können, wird in Leitbildern von Pflegeeinrichtungen mit traditionellem Wohnbereich die Pflege-Charta beschrieben. In anderen Leitbildern von Pflegeeinrichtungen mit einem Demenzwohnbereich und von Pflegeeinrichtungen mit einem traditionellen Wohnbereich wird auf die Variation von Menschenbildern eingegangen, darunter das humanistische Menschenbild oder das christliche Menschenbild.

In Leitbildern von Pflegeeinrichtungen beider Wohnbereichstypen werden unterschiedliche Aspekte, die die Haltung gegenüber der/des Bewohner:in beschreiben, benannt. Diese Aspekte sind als Grundsätze formuliert, die z. T. auch als Voraussetzungen für eine erfolgreiche Versorgung und den gemeinsamen Umgang benannt werden. Zu Aspekten, die Haltung beschreiben, zählen u. a. Engagement, Gleichberechtigung, Empathie; Respekt, Wertschätzung, Ehrlichkeit, Vertrauen und Loyalität.

In den Leitbildern von Pflegeeinrichtungen beider Wohnbereichstypen wird außerdem der Anspruch an die Atmosphäre in der Pflegeeinrichtung beschrieben. In Leitbildern von Pflegeeinrichtungen mit traditionellem Wohnbereich wird der Anspruch an ein harmonisches Betriebsklima, an eine Atmosphäre des Vertrauens oder an eine Atmosphäre der Menschlichkeit formuliert. In Leitbildern von Pflegeeinrichtungen mit Demenzwohnbereich ist es der Anspruch, eine Atmosphäre mit Wohlfühlcharakter zu erzielen oder eine Atmosphäre der Sicherheit und Geborgenheit für die/den Bewohner:in zu schaffen, z. B.:

„Deshalb sorgen wir dafür, dass sich unsere Bewohner in einer Atmosphäre der Sicherheit und Geborgenheit rundum wohl fühlen.“ (Pflegeeinrichtung 54, Demenzwohnbereich)

## Diskussion

Das Ziel dieser Studie war es, mithilfe einer Dokumentenanalyse Leitbilder verschiedener Pflegeeinrichtungen in Deutschland dahingehend zu untersuchen, wie person-zentrierte Versorgung beschrieben wird, und ob es Unterschiede zwischen Leitbildern von Pflegeeinrichtungen mit Demenzwohnbereich und traditionellen Wohnbereichen gibt.

Die analysierten Leitbilder adressieren Aspekte, die dem Ansatz der person-zentrierten Versorgung zugeordnet werden können [8, 18]. Insbesondere Leitbilder von Pflegeeinrichtungen mit Demenzwohnbereichen beschreiben eine Kombination zentraler Aspekte der person-zentrierten Versorgung, die Leitbilder von Pflegeeinrichtungen mit einem traditionellen Wohnbereich nicht beschreiben. Dazu zählt, dass ausschließlich in Leitbildern von Pflegeeinrichtungen mit Demenzwohnbereich die/der Bewohner:in als Person verstanden wird, während gleichzeitig person-zentrierte Aspekte, wie z. B. das person-zentrierte Handeln beschrieben werden. Leitbilder von Pflegeeinrichtungen mit Demenzwohnbereich haben einen stärkeren Fokus auf die Ermöglichung und den Erhalt von Autonomie und Selbstbestimmung der/des Bewohner:in. In Leitbildern, die die/den Bewohner:in nicht als Person definieren, wird häufig eine Variation verschiedener Verständnisse bzw. Konzepte deutlich. Dieses Phänomen des Eklektizismus – die Kombination verschiedener Konzepte als auch deren variable Auslegung in Leitbildern – wurde auch in der Leitbildanalyse von Kalis [16] deutlich.

Unsere Erkenntnisse lassen offen, wie die Pflegeeinrichtungen, deren Leitbilder wir analysierten, den person-zentrierten Ansatz implizit für ihr Haus übersetzen. Die Leitbilder wurden zu einem Großteil vor der Veröffentlichung des Expertenstandards zur Beziehungsgestaltung in der Pflege von Menschen mit Demenz im Jahr 2019 [10] erstellt. Daher ist anzunehmen, dass sich Pflegeeinrichtungen, die in ihren Leitbildern eine Kombination zentraler Aspekte der person-zentrierten Versorgung adressieren bzw. den person-zentrierten Ansatz nach Kitwood explizit benennen, sich insbesondere aktiv mit dem breit bekannten und im deutschsprachigen Raum oft zitierten person-zentrierten Ansatz nach Kitwood [18] beschäftigt haben. Dennoch kann sich die Transferleistung theoretischer Konzepte in die Pflegepraxis als herausfordernd gestalten, wenn sich Pflegeeinrichtungen zuvor auf andere Ansätze, wie z. B. das Modell der fördernden Prozesspflege [20], in denen nicht explizit person-zentrierte Versorgung von Menschen mit Demenz im Fokus steht, fokussiert haben.

Die variable Auslegung konzeptioneller Aspekte in verschiedenen Leitbildern erweckt daher die Frage, wie person-zentrierte Versorgung in diesen Pflegeeinrichtungen gelebt wird. Aus unseren Ergebnissen wird deutlich, dass die Leitbilder häufig durch Personen des Qualitätsmanagements oder der Leitungsebene erstellt wurden. Unklar ist hierbei, inwiefern Mitarbeitende in den Prozess der Erstellung der jeweiligen Leitbilder einbezogen wurden. Kelleter [17] betont, dass entgegen eines Top-down-Ansatzes alle Akteur:innen in die Leitbildentwicklung und -erarbeitung eingebunden werden und Leitbilder im fortlaufenden Prozess reflektieren sollen, damit sich die Pflegeeinrichtung im Sinne ihrer Corporate Identity mit dem Inhalt der Leitbilder identifizieren und diese auch leben können. Neben dem Inhalt der Leitbilder spielt auch die Nutzung von Sprache eine wichtige Rolle [9]. In einigen analysierten Leitbildern wurde die z. T. fehlende Nutzung von alters- und demenzsensitiver Sprache deutlich. Hierbei wurden Defizite und Erkrankungen der Bewohner:innen durch Formulierungen wie „alte Menschen“ (Pflegeeinrichtung 83, traditioneller Wohnbereich), „verwirrte Bewohner“ (Pflegeeinrichtung 23, traditioneller Wohnbereich), „Demenzkranke“ (Pflegeeinrichtung 63, Demenzwohnbereich) sprachlich in den Vordergrund gesetzt. Um negative Stereotypen zu überwinden und eine Haltung des Respekts und der Wertschätzung gegenüber Menschen im Alter und Menschen mit Demenz einzunehmen, braucht es eine Sprache, die diese Haltung widerspiegelt [2, 11].

Die Ergebnisse der vorliegenden Leitbildanalyse lassen darauf schließen, dass Leitbilder von Pflegeeinrichtungen mit einem Demenzwohnbereich vermehrt person-zentriere Aspekte beinhalten im Vergleich zu Pflegeeinrichtungen mit einem traditionellen Wohnbereich. Vor dem Hintergrund, dass Menschen mit Demenz die größte Bewohner:innengruppe in Pflegeeinrichtungen repräsentieren [14], ist grundsätzlich eine person-zentrierte Versorgung von Relevanz, die auch in den Leitbildern adressiert werden sollte. Der Expertenstandard zur Beziehungsgestaltung in der Pflege von Menschen mit Demenz definiert den person-zentrierten Ansatz als konzeptionelle Basis. Um herauszufinden, ob und inwiefern dieser Expertenstandard auf die Erstellung und Entwicklung von Leitbildern in Pflegeeinrichtungen Einfluss haben könnte, bedarf es eines Vergleichs von Leitbildern, die vor und nach der Veröffentlichung des Expertenstandards *Beziehungsgestaltung in der Pflege von Menschen mit Demenz* im Jahr 2019 [10] erstellt und implementiert wurden.

## Schlussfolgerungen

- In der Analyse der Leitbilder wurde eine Variation unterschiedlicher normativ ausgeprägter Verständnisse von person-zentrierter Versorgung deutlich. Ein einheitliches gelebtes Grundverständnis unter den Mitarbeitenden kann insofern in diesen Pflegeeinrichtungen hinterfragt werden. Um ein Leitbild person-zentriert ausrichten zu können, bedarf es einer umfassenden Auseinandersetzung mit dem person-zentrierten Ansatz.
- Vereinzelte Leitbilder beschreiben eine Kombination zentraler person-zentrierter Aspekte. Dies bestärkt den internationalen Konsens darüber, dass person-zentrierte Versorgung als Bestandteil in Leitbildern übernommen wird [24].
- Die Umsetzung von Leitbildern im Praxisalltag sollte in den Blick genommen werden. Sonst besteht die Gefahr, dass Leitbilder in erster Linie ein Managementinstrument mit begrenztem praktischen Wert sind und nur geringen bis keinen Einfluss auf den Versorgungsalltag in Pflegeeinrichtungen haben [9].

## Data Availability

Der Zugang zu den Daten ist auf Anfrage bei dem Deutschen Zentrum für Neurodegenerative Erkrankungen (DZNE) e. V., Standort Witten, möglich. Bitte kontaktieren Sie das Datenmanagement des Deutschen Zentrums für Neurodegenerative Erkrankungen (DZNE) e. V., Standort Witten (data-management-witten(at)dzne.de).
